# Association of Hypoxic Burden With Cardiovascular Events: A Risk Stratification Analysis of the Randomized Intervention With CPAP in Coronary Artery Disease and Sleep Apnea Cohort

**DOI:** 10.1016/j.chest.2025.07.4081

**Published:** 2025-08-14

**Authors:** Yüksel Peker, Yeliz Celik, Andrey Zinchuk, Scott A. Sands, Susan Redline, Ali Azarbarzin

**Affiliations:** a Department of Pulmonary Medicine, Koc University School of Medicine, and Koc University Research Center for Translational Medicine, Istanbul, Türkiye; b Division of Sleep and Circadian Disorders, Brigham and Women's Hospital & Harvard Medical School, Boston, MA; c Division of Pulmonary, Allergy, and Critical Care Medicine, University of Pittsburgh School of Medicine, Pittsburgh, PA; d Department of Molecular and Clinical Medicine, Institute of Medicine, Sahlgrenska Academy, University of Gothenburg, Gothenburg, Sweden; e Department of Clinical Sciences, Respiratory Medicine and Allergology, Faculty of Medicine, Lund University, Lund, Sweden; f Department of Internal Medicine, School of Medicine, Yale University, New Heaven, CT

**Keywords:** apnea-hypopnea index, cardiovascular outcomes, coronary artery disease, hypoxic burden, OSA

## Abstract

**Background:**

The apnea-hypopnea index (AHI), the standard measure of OSA, has limitations in reflecting disease severity.

**Research Question:**

Is high hypoxic burden (HB) more strongly associated with major cardiovascular and cerebrovascular adverse events (MACCEs) than AHI of ≥ 30 events/h?

**Study Design and Methods:**

This secondary analysis of the Randomized Intervention With CPAP in Coronary Artery Disease and Sleep Apnea observational cohort included 368 adults with OSA (AHI ≥ 15 events/h) with (n = 155) and without (n = 244) excessive daytime sleepiness (EDS), defined as an Epworth Sleepiness Scale score of ≥ 10. HB was calculated as the total area under respiratory event-related desaturations divided by total sleep time. Patients were classified as having high or low HB based on the median (60.7%min/h). The primary outcome was the incident of the first MACCE. Cox proportional hazard models assessed associations in the full cohort and by CPAP allocation and adherence (nonadherent or no positive airway pressure [PAP] group, n = 262; adherent [adjusted PAP use ≥ 4 h/night for all nights at 1-year follow-up], n = 106). In an exploratory analysis, participants were grouped into 4 categories based on median AHI and HB (low and low, low and high, high and low, and high and high, respectively).

**Results:**

Over a median follow-up of 4.7 years, high HB was associated with MACCEs (adjusted hazard ratio, 1.87; 95% CI, 1.17-2.98; *P* = .009), particularly among untreated or nonadherent patients and those with baseline EDS. AHI of ≥ 30 events/h was not associated significantly with MACCEs (*P* = .366). When modelled continuously, HB and AHI each were associated with MACCEs; however, compared with low AHI and low HB, only high HB, regardless of AHI level, was linked to increased risk. In contrast, high AHI and low HB was not associated with MACCEs.

**Interpretation:**

High HB, but not AHI of ≥ 30 events/h, was associated with MACCEs in adults with moderate to severe OSA. Although AHI was associated with outcomes when modelled continuously, elevated risk seemed to be driven primarily by high HB.

**Clinical Trial Registration:**

ClinicalTrials.gov; No.: NCT00519597; URL: www.clinicaltrials.gov

## Graphical Abstract

**Figure:**
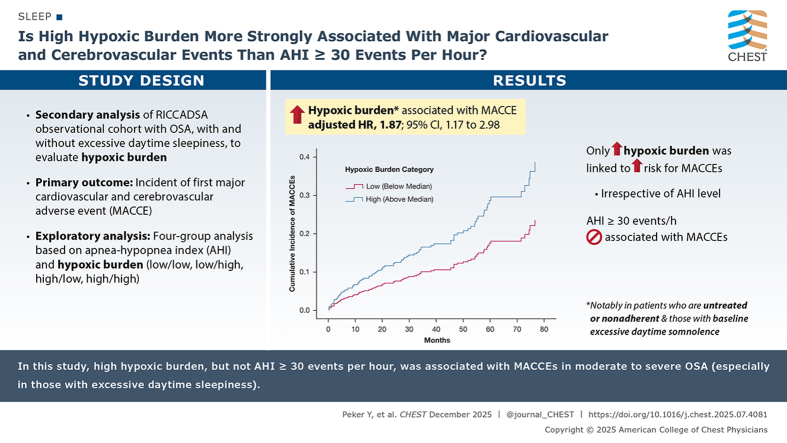

**Take-Home Points**

**Study Question:** Does hypoxic burden (HB) better predict major cardiovascular and cerebrovascular events (MACCEs) than apnea-hypopnea index (AHI)?

**Results:** High HB at baseline predicted MACCEs in patients with moderate to severe OSA with an adjusted hazard ratio 1.87 (95% CI, 1.17-2.98; *P = .*009), whereas AHI of ≥ 30 events/h at baseline was not associated with MACCEs (*P = .*366).

**Interpretation:** Randomized controlled trials are needed to assess whether targeting those with high HB improves the outcomes of positive airway pressure therapy.

Coronary artery disease (CAD) is associated with increased morbidity and mortality worldwide, despite advances in pharmacologic therapy and revascularization procedures. OSA, characterized by intermittent periods of upper airway obstruction and recurrent hypoxemia, is highly prevalent in adults with CAD (50%, as compared with 10%-20% in the general adult population),^1^ and early clinical cohort studies showed that patients with coexisting CAD and OSA had worse prognosis than the patients with CAD alone.^2^^,^^3^ CPAP, the gold standard treatment for OSA, has been shown to reduce excessive daytime sleepiness (EDS) and to improve quality of life in symptomatic patients with OSA.^4^ However, most patients with OSA are asymptomatic, and treatment is offered to them to reduce the risk of cardiovascular outcomes. In OSA without EDS, the randomized controlled trials (RCTs) Randomized Intervention With CPAP in Coronary Artery Disease and Sleep Apnea (RICCADSA),^5^ Sleep Apnea Cardiovascular Endopints (SAVE),^6^ and Impact of Sleep Apnea Syndrome in the Evolution of Acute Coronary Syndrome (ISAACC)^7^ have not shown any reduction in major cardiovascular and cerebrovascular events (MACCEs) in intention-to-treat analyses. Several reasons have been proposed for the neutral results, including poor adherence to CPAP therapy in those trials and that they largely excluded individuals with EDS.^8^ The widely accepted adherence level for CPAP use is at least 4 h/d and 70% of the days per period, which corresponds 2.8 h/d for all days^9^ in sleep clinic cohorts. The 4-hour cutoff level on nights used showed significant benefit of CPAP in the RICCADSA trial in the post hoc on-treatment analysis of the patients without EDS.^5^ Although the secondary individual patient data meta-analysis of these trials and on-treatment analysis suggested the significant effect of CPAP adherence with cutoff of 4 h/night for all nights, these studies are limited by confounding healthy-user effect.^10^ Moreover, a recent secondary analysis of the RICCADSA trial revealed that patients with CAD as well as OSA and EDS receive even less cardiovascular benefit from positive airway pressure (PAP) than the patients without EDS.^11^

Another explanation for the null findings of previous trials is substantial heterogeneity in OSA, which is not captured by the apnea-hypopnea index (AHI).^12^ Previous trials enrolled participants based on AHI without any consideration for the level of hypoxemia or other important disease-characterizing features.^12^^,^^13^ For example, novel markers of OSA-related disease severity, including hypoxic burden (HB)^14^ and cardiac autonomic response,^15^ have been suggested as strong predictors of adverse cardiovascular health outcomes. Heart rate response to apneas^15^ as well as HB recently was suggested as a predictor of cardiovascular benefit of treating OSA with CPAP.^16^

In the current study, we aimed to assess whether the magnitude of the association of HB with MACCEs is greater than that of AHI of ≥ 30 events/h in patients with CAD and moderate to severe OSA in the RICCADSA cohort. Exploratory analyses compared the risk of MACCEs in 4 groups created by median AHI and HB.

## Study Design and Methods

### Study Design, Participants, Sleep Studies, Group Assignment, and Follow-Up

This was a secondary analysis of an observational study involving participants from the RICCADSA cohort. Details of the study design and methods of the parent trial have been published previously^5^^,^^17^^,^^18^ and are described in detail in e-Appendix 1. In brief, the entire study cohort included adult patients with angiography-verified CAD who had undergone revascularization by percutaneous coronary intervention or coronary artery bypass grafting within 6 months before the baseline sleep study. Inclusion criteria for the RCT arm comprised an AHI of ≥ 15 events/h with the home sleep apnea test (HSAT) for OSA diagnosis, and the absence of EDS, defined by an Epworth Sleepiness Scale (ESS) score of < 10 at baseline. A concurrent observational arm consisted of patients with an AHI of > 15 events/h and an ESS score of ≥ 10 as well as patients without OSA defined as an AHI of < 5 events/h with the HSAT. All patients with OSA underwent overnight polysomnography recording before the study start.

**Figure 1:**
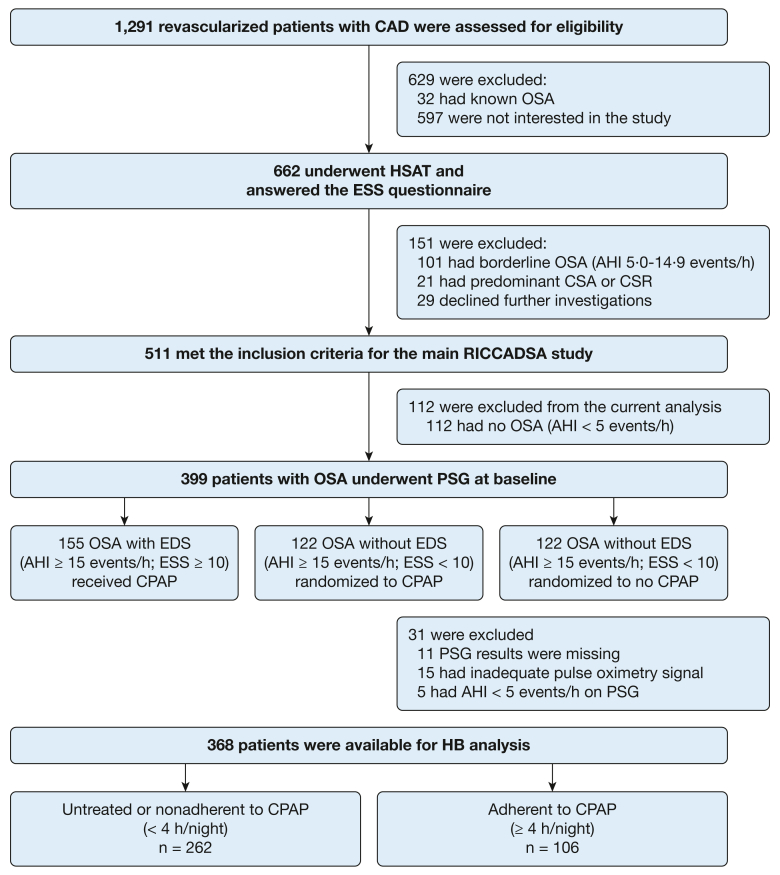
Flow diagram showing progression of patients through the study. AHI = apnea-hypopnea index; CAD = coronary artery disease; CSA = central sleep apnea; CSR = Cheyne Stokes respiration; ESS = Epworth Sleepiness Scale; HB = hypoxic burden; HSAT = home sleep apnea test; PSG = polysomnography; RICCADSA = Randomized Intervention With CPAP in Coronary Artery Disease and Sleep Apnea.

The current study included 399 participants comprising 244 participants from the RICCADSA RCT (ESS score < 10) and 155 participants from the CPAP observational arm (ESS score > 10). The final analytic sample included all 368 patients with OSA who had available polysomnography data with adequate quality pulse oximetry signals (Fig 1). The analyses were stratified by the treatment groups: 262 participants who either were untreated (randomized to no CPAP) or nonadherent (adjusted CPAP use < 4 h/night for all nights at the 1-year follow-up) and 106 patients adherent to CPAP (adjusted CPAP use ≥ 4 h/night for all nights), respectively. The study participants were recruited between December 2005 and November 2010, and the follow-up was completed in May 2013.

The study protocol was approved by the ethics committee of the Medical Faculty of the University of Gothenburg (Identifier: 207-05, September 13, 2005; amendment T744-10, November 26, 2010; amendment T512-11, June 16, 2011), and all patients provided written informed consent. The RICCADSA trial was registered with ClinicalTrials.gov (Identifier: NCT00519597) as well as with the national researchweb.org (FoU i Sverige—Research and Development in Sweden; Identifier: VGSKAS-4731, April 29, 2005).

### Sleep Studies

See e-Appendix 1 for further information.

#### Home Sleep Apnea Test

The portable HSAT was performed with the Embletta Portable Digital System device (Embla). A nasal pressure transducer along with the thoracoabdominal respiratory inductance bands were used to measure airflow and to monitor respiratory efforts. A finger pulse oximeter was used to measure oxyhemoglobin saturation. Apneas and hypopneas were scored based on the Chicago criteria.^19^

#### Polysomnography

All patients with CAD and a diagnosis of OSA based on the HSAT underwent unattended overnight polysomnography (Embla A10; Embla) in the hospital. Polysomnography recordings were scored by an observer masked to clinical data and baseline screening results from the HSATs. Obstructive events on the polysomnography results were scored according to the same apnea and hypopnea criteria applied for the HSAT recordings.

### Hypoxic Burden

The calculation of the HB followed methods previously described.^14^ Briefly, HB was defined as the total area under the respiratory event-related desaturation curve divided by the sleep duration. The area calculation was from a pre-event baseline defined as maximum value of oxygen saturation during a 100-second interval before the end of each respiratory event, as described previously.^14^ The area calculation was guided by a patient-specific search window, obtained from ensemble averaging of all individual oxygen saturation signals aligned with respect to end of respiratory events.^14^ Patients were categorized as having a high or a low baseline hypoxic burden according to the median value (60.7%min/h). All HB measurements were calculated from the polysomnography results.

### Outcomes and the Study Oversight

The primary end point was the composite of repeat revascularization, myocardial infarction, stroke, and cardiovascular mortality from the study start. Only the first event was calculated in the combined end point for participants who experienced > 1 event during the follow-up period. As previously described,^5^ an independent clinical event committee reviewed all data obtained from patient records and death certificates by the end of May 2013, masked to the group allocation. A data monitoring board monitored a random 10% selection of the database for clinical data and follow-up, including CPAP use and primary composite end points.

### Statistical Analysis

Descriptive statistics were reported as mean (SD) or as median (interquartile range [IQR]) for continuous variables and as frequencies and percentages for categorical variables. Two-sided χ^2^ tests and *t* tests were applied to describe balance between groups. Complete-case analysis was applied for the missing data.

CPAP use was evaluated as average use hours per night for nights used at 12 months after the study start (unadjusted use), as a percentage of nights during the period up to 12 months, and as hours per night multiplied by the percentage of CPAP nights as the adjusted use, corresponding CPAP hours per night for all nights, using data downloaded from the CPAP devices.

The patients with OSA with low vs high HB were compared in the entire cohort and in 2 subgroups: (1) untreated (randomized to no CPAP) or nonadherent patients (adjusted CPAP use < 4 h/night) and (2) CPAP-adherent patients (adjusted CPAP use at least 4 h/night) at the 12-month follow-up.

Associations between high HB as well as AHI of ≥ 30 events/h and MACCEs were tested by Cox proportional hazards regression adjusted for age, sex, BMI, type of revascularization at baseline, diabetes, former revascularization, current smoking at baseline, hypertension, history of atrial fibrillation, acute myocardial infarction at baseline, EDS, allocation to CPAP treatment in the entire cohort, total sleep time, and baseline low-density lipoprotein levels. The proportional hazard assumption was tested regarding the interaction between the time and the covariates in the model. For the subgroup analysis, age, sex, BMI, and only the variables that were significant in the entire cohort were included as covariates. In addition and similar to prior studies,^14^ to test the association of continuous HB and MACCE, HB was log-transformed. The association of continuous AHI with MACCE also was tested. Univariable and multivariable Cox regression models were performed with a 5% significance level. Kaplan-Meier survival curves were plotted to illustrate incident MACCEs per HB categories as well as AHI categories stratified by the treatment groups. A subgroup analysis was conducted for the patients with vs without EDS. Moreover, to compare HB and AHI, these 2 variables were categorized by their median values, and 4 groups were created: low AHI and low HB, low AHI and high HB, high AHI and low HB, and high AHI and high HB. In additional sensitivity analyses, we added both AHI and HB as continuous and categorical (median or higher vs less than median) variables in the same model and reported the hazard ratios (HRs). All statistical analyses were performed using IBM SPSS Statistics for Windows version 28.0 (IBM Corp.)

## Results

### Study Participants

In all, 368 participants (mean [SD] age, 64.7 [8.1] years; 319 male sex [86.7 %]) were eligible for the current protocol, excluding the patients with missing polysomnography findings (Fig 1). The median value of the HB was 60.7%min/h (35.1%min/h-111.8%min/h) in the entire cohort. Baseline characteristics of the untreated and nonadherent patients with OSA and adherent patients with OSA were similar except that the ESS scores that were higher in the adherent group, both in high-HB vs low-HB subgroups (Table 1). Median adjusted CPAP use was 0.0 h/night (IQR, 0.0-1.0 h/night) in the untreated and nonadherent group and 6.1 h/night (IQR, 5.0-6.9 h/night) at the 1-year follow-up in the adherent group (*P < .*001). Median HB was similar in the untreated and nonadherent subgroups in both low-HB and high-HB subgroups (Table 2).

**Table 1:** Demographic and Baseline Clinical Characteristics of the Patients With CAD With OSA in the RICCADSA Cohort (n = 368)

Variable	Untreated of Nonadherent to CPAP (< 4 h/night; n = 262)		Adherent to CPAP (≥ 4 h/night; n = 106)	
	OSA With Low HB (n = 136)	OSA With High HB (n = 126)	OSA With Low HB (n = 48)	OSA With High HB (n = 58)
Age, y	63.8 (59.0-69.7)	66.1 (59.5-72.9)	66.6 (60.2-71.3)	64.4 (60.0-69.3)
Male sex	85.3	87.3	83.3	91.4
BMI, kg/m^2^	28.4 (26.0-30.2)	29.0 (26.8-31.6)	27.6 (25.8-30.8)	29.4 (25.7-32.9)
Obesity	28.7	37.3	29.2	44.8
ESS score	7.0 (4.0-10.0)	8.0 (4.0-11.0)	10.0 (6.0-13.0)	10.0 (6.0-11.0)
EDS, ESS ≥ 10	28.1	38.1	56.3	55.2
Current smoking	16.2	19.0	12.5	13.8
CABG at baseline	22.8	31.0	18.8	29.3
Former revascularization	19.9	25.4	20.8	15.5
Hypertension	56.6	64.3	60.4	62.1
AMI at baseline	47.1	46.0	47.9	62.1
History of atrial fibrillation	12.5^a^	26.2^a^	14.6	17.2
Diabetes mellitus	23.5	23.0	20.8	32.8
History of stroke	7.5	9.5	6.3	6.9
Lung disease	6.6	11.9	8.3	3.4
Severe OSA, AHI ≥ 30 events/h	27.2^a^	92.9^a^	29.2^a^	94.8^a^

Data are presented as percentage or median (interquartile range). AHI = apnea hypopnea index; AMI = acute myocardial infarction; CABG = coronary artery bypass grafting; CAD = coronary artery disease; EDS = excessive daytime sleepiness; ESS = Epworth Sleepiness Scale; HB = hypoxic burden; RICCADSA = Randomized Intervention With CPAP in Coronary Artery Disease and Sleep Apnea.

a *P* < .05.

**Table 2:** Results of the Baseline Polysomnography Recordings in the Hospital in the Analytic Sample

Variable	Untreated or Nonadherent to CPAP (< 4 h/night; n =262)		Adherent to CPAP (≥ 4 h/night; n = 106)	
	OSA With Low HB (n =136)	OSA With high HB (n =126)	OSA With Low HB (n = 48)	OSA With High HB (n =58)
HB, %min/h	34.6 (23.6-45.7)^a^	113.8 (86.9-164.9)^a^	37.8 (25.4-49.0)^a^	110.0 (79.2-172.3)^a^
TST, min	393.2 (320.0-456.5)^a^	380.8 (315.1–432.1)^a^	420.8 (370.6-474.1)	388.0 (336.8-431.5)
Sleep onset, min	9.8 (5.7-18.1)	11.4 (6.8-18.8)	8.7 (5.0-15.3)^a^	11.2 (7.0-23.3)^a^
Sleep efficiency, %	81.1 (70.6-87.4)	78.5 (68.2-86.4)	84.4 (72.8-91.5)^a^	78.1 (72.2-85.9)^a^
Slow-wave sleep, min	26.8 (3.6-52.9)^a^	11.8 (0.0-40.8)^a^	35.0 (0.0-58.8)^a^	11.3 (0.0-21.6)^a^
Slow-wave sleep %, % of TST	7.6 (1.1-14.2)^a^	3.0 (0.0-9.4)^a^	7.1 (0.0-14.7)^a^	2.5 (0.0-5.6)^a^
REM sleep, min	54.5 (31.1-74.0)^a^	39.8 (23.3-63.5)^a^	67.8 (48.0-81.5)^a^	39.3 (24.6-59.3)^a^
REM sleep %, % of TST	14.4 (9.6-17.8)^a^	10.4 (7.0-15.3)^a^	15.2 (12.1-18.8)^a^	10.2 (7.4-14.9)^a^
Arousal index, events/h	38.2 (29.0-49.9)^a^	60.6 (48.1-75.1)^a^	40.1 (29.5-51.4)^a^	64.2 (45.7-76.9)^a^
AHI, events/h	23.2 (15.3-31.1)^a^	56.0 (43.3-72.3)^a^	23.3 (16.0-30.5)^a^	54.9 (40.7-70.3)^a^
REM AHI, events/h	26.3 (16.8-50.9)^a^	53.9 (35.1-68.8)^a^	35.3 (17.8-51.2)^a^	58.5 (41.9-67.6)^a^
Non-REM AHI, events/h	22.3 (13.2-30.0)^a^	59.1 (40.9-74.1)^a^	22.5 (13.1-30.3)^a^	55.5 (40.5-70.1)^a^
Supine time, min	93.9 (37.4-169.3)^a^	141.2 (70.0-229.1)^a^	103.1 (43.0-257.0)	152.4 (76.0-231.1)
Supine time %, % of TST	23.9 (9.9-45.2)^a^	40.6 (18.8-65.6)^a^	30.2 (11.7-57.0)	43.5 (22.0-65.0)
Supine AHI, events/h	41.1 (26.7-65.7)^a^	66.5 (52.2-79.3)^a^	42.7 (27.9-61.8)^a^	64.0 (51.6-80.5)^a^
ODI, events/h	9.1 (5.9-15.0)^a^	31.3 (22.4-45.5)^a^	11.0 (7.4–17.2)^a^	29.2 (21.4-45.5)^a^
Average Spo_2_, %	94.3 (93.2-95.2)^a^	93.4 (92.7-94.4)^a^	93.9 (92.9-95.1)	93.4 (92.5-94.8)
Nadir Spo_2_, %	86.0 (83.0-88.0)^a^	81.0 (76.0-84.0)^a^	86.0 (81.3-88.0)^a^	80.0 (76.0-85.0)^a^
Spo_2_ < 90%, % of TST	0.4 (0.1-1.7)^a^	4.3 (1.1-10.4)^a^	0.6 (0.1-2.6)^a^	5.6 (1.1-9.5)^a^
Heart rate, beats/min	57.5 (52.6-62.6)	59.4 (54.5-67.2)	56.9 (50.2-63.0)	57.9 (51.5-65.5)

Data are presented as percentage or median (interquartile range). AHI = apnea hypopnea index; HB = hypoxic burden; ODI = oxygen desaturation index; REM = rapid eye movement; Spo_2_ = oxygen saturation; TST = total sleep time.

a *P* < .05.

### Baseline Characteristics of the Untreated and Nonadherent Patients

As shown in Table 1, among the untreated and nonadherent patients, those with high HB compared with low HB had slightly higher BMI and included a higher proportion of individuals with a history of atrial fibrillation as well as severe OSA (ie, AHI ≥ 30 events/h), whereas other characteristics did not differ significantly. As shown in Table 2, the patients with high HB had slightly shorter total sleep time, significantly lower proportion of slow-wave sleep and rapid eye movement (REM) sleep, and higher total AHI, REM-AHI, and supine-AHI values as well as more impaired oxygenation indexes as expected.

### Baseline Characteristics of the Adherent Patients

As illustrated in Table 1, among the treated and adherent participants, all the baseline characteristics were similar between the low-HB and high-HB subgroups except the proportion of patients with severe OSA (AHI ≥ 30 events/h), which, as expected, was significantly higher in the high-HB subgroup. Regarding the polysomnography characteristics, the high-HB subgroups had shorter sleep onset time and lower sleep efficiency and proportion of slow-wave sleep and REM sleep than did the patients with low HB at baseline (Table 2). As expected, total AHI, REM-AHI, and supine-AHI values were higher and oxygenation indices were more severe among the high-HB subgroup.

### Outcomes

**Figure 2:**
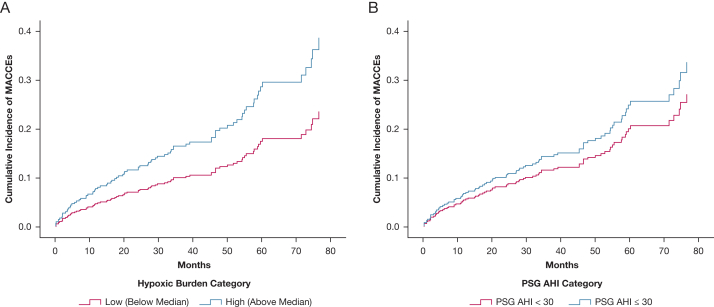
A, B, Kaplan-Meier curves showing the cumulative incidences of MACCEs in the entire cohort of patients with OSA with low vs high hypoxic burden at baseline (A) and the cumulative incidences of MACCEs in patients with AHI of < 30 events/h vs AHI of ≥ 30 events/h (B). AHI = apnea-hypopnea index; MACCE = major adverse cardiovascular and cerebrovascular event; PSG = polysomnography.

Median follow-up to the first MACCE or the end of study was 57.0 months (IQR, 43.0-70.0 months). Of the 368 patients analyzed, 81 patients (22.0%) reached the end point, including 31 patients (16.8%) in the low-HB group and 50 patients (27.2%) in the high-HB group (Fig 2A). The proportional HR assumptions were satisfied for each independent variable in the Cox model (all *P* > .05). In the adjusted model, high HB was associated with MACCEs with an HR of 1.87 (95% CI, 1.17-2.98; *P = .*009) accounting for age, sex, BMI, type of revascularization at baseline, diabetes, former revascularization, current smoking at baseline, hypertension, history of atrial fibrillation, acute myocardial infarction at baseline, EDS, and allocation to CPAP treatment (Table 3). Adding total sleep time and baseline low-density lipoprotein levels as covariates in the model did not change the results meaningfully; high HB remained associated significantly with MACCE (HR, 1.79; 95% CI, 1.12-2.86; *P = .*015). As shown in Figure 2B and Table 3, AHI of ≥ 30 events/h was not associated significantly with the MACCEs (HR, 1.33; 95% CI, 0.83-2.14; *P = .*232). Further analysis of the continuous variables revealed that HB and AHI each were associated with the MACCEs in the fully adjusted model when tested separately (HR, 1.26 [95% CI, 1.00-1.59; *P = .*049] vs HR, 1.28 [95% CI, 1.02-1.59; *P = .*030] per 1 SD).

**Table 3:** Variables Associated With MACCEs in Patients With CAD and OSA in the Entire RICCADSA Cohort (n = 368)

Variable	Adjusted HR	95% CI	*P* Value
Model I			
High vs low HB	1.87	1.17-2.98	.009
Age	1.02	0.99-1.05	.231
Female vs male sex	0.74	0.35-1.55	.423
BMI	0.96	0.90-1.03	.248
Diabetes mellitus	1.74	1.04-2.91	.035
Former revascularization	2.28	1.42-3.65	< .001
CABG vs PCI	0.33	0.16-0.69	.003
Hypertension	1.52	0.93-2.49	.095
Current smoking	1.99	1.12-3.55	.019
History of atrial fibrillation	1.02	0.52-2.00	.944
Acute myocardial infarction	1.06	0.66-1.70	.804
Allocation to CPAP treatment	1.12	0.68-1.83	.662
Model II			
AHI ≥ 30 events/h vs < 30 events/h	1.33	0.83-2.14	.232
Age	1.02	0.99-1.05	.150
Female vs male sex	0.71	0.33-1.49	.357
BMI	0.97	0.91-1.04	.396
Diabetes mellitus	1.71	1.03-2.85	.039
Former revascularization	2.30	1.44-3.70	< .001
CABG vs PCI	0.35	0.17-0.72	.005
Hypertension	1.59	0.97-2.60	.068
Current smoking	2.03	1.14-3.61	.016
History of atrial fibrillation	1.10	0.57-2.14	.777
Acute myocardial infarction	1.11	0.69-1.77	.672
Allocation to CPAP treatment	1.10	0.67-1.80	.708

AHI = apnea-hypopnea index; CABG = coronary artery bypass grafting; CAD = coronary artery disease; HB = hypoxic burden; HR = hazard ratio; MACCE = major adverse cardiovascular and cerebrovascular event; PCI = percutaneous coronary intervention; RICCADSA = Randomized Intervention With CPAP in Coronary Artery Disease and Sleep Apnea.

In a further analysis of the 262 patients in the untreated and nonadherent group, 58 patients reached the end point (22.1%), including 23 patients (16.9%) in the low-HB subgroup, and 35 patients (27.8%) in the high HB subgroup. In a multivariable model, high HB predicted the MACCEs with an HR of 1.87 (95% CI, 1.08-3.22; *P = .*024) after adjusting for age, sex, BMI, revascularization type, diabetes status, current smoking, and former revascularization. In the subgroup analysis, restricted to the untreated patients, the unadjusted HR was 1.15 (95% CI, 0.52-2.52; *P = .*731) among 113 patients randomized to no CPAP. Of the 106 patients analyzed in the adherent group, 23 patients reached the end point (21.7%), including 8 patients (16.7%) in the low-HB subgroup and 15 patients (25.9%) in the high-HB subgroup. No significant association of high HB with the MACCEs was found in the multivariable analysis (HR, 1.80; 95% CI, 0.74-4.34; *P = .*549).

**Figure 3:**
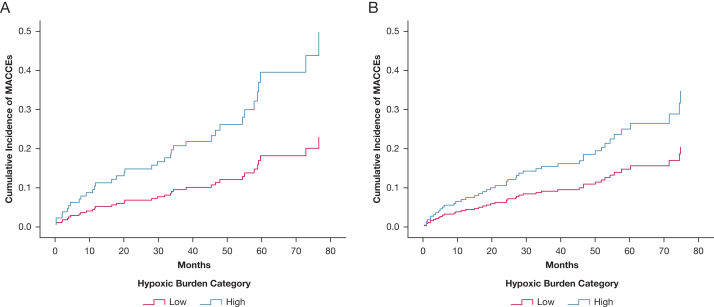
A, B, Kaplan-Meier curves showing the cumulative incidences of MACCEs among the patients with OSA with excessive daytime sleepiness at baseline (A) and the cumulative incidences of MACCEs among the patients with OSA without excessive daytime sleepiness at baseline (B). MACCE = major adverse cardiovascular and cerebrovascular event.

As shown in Figure 3A, the significant association between the high HB and MACCEs was prevalent among the patients with moderate to severe OSA with EDS (n = 146) in the entire cohort with an HR of 2.18 (95% CI, 1.03-4.63; *P = .*043), whereas a tendency toward a significant association among the patients without EDS (n = 222) was noted (HR, 1.70; 95% CI, 0.92-3.13; *P = .*089) (Fig 3B).

When comparing HB and AHI categorized by their median values (median HB, 60.7%min/h vs median AHI, 36.1 events/h), compared with individuals with low AHI and low HB, only groups with high HB were at increased risk of MACCE, whereas individuals with high AHI but low HB were not (Table 4). Finally, in additional sensitivity analyses, when both HB and AHI were added to the same model as continuous variables, both metrics showed nonsignificant associations with the primary end point (HB: fully adjusted HR for per 1-SD increase, 1.12 [95% CI, 0.80-1.56; *P* = .522]; AHI: HR per 1-SD increase, 1.18 [95% CI, 0.84-1.65; *P* = .344]). However, when they were modeled together as categorical variables, only HB showed a significant association with the primary end point (HB: fully adjusted HR, 2.44 [95% CI, 1.22-4.87; *P* = .012]; AHI: HR, 0.70 [95% CI, 0.36-1.38; *P* = .309]).

**Table 4:** Association of HB and AHI With MACCEs by their Median Values in Patients With CAD and OSA in the RICCADSA Cohort (n = 368)

Variable	Adjusted HR	95% CI	*P* Value
Low HB (< 60.7%min/h) vs low AHI (< 36.1 events/h)	1	NA	NA
Low HB (< 60.7%min/h) vs high AHI (≥ 36.1 events/h)	0.82	0.24-2.75	.745
High HB (≥ 60.7%min/h) vs low AHI (< 36.1 events/h)	2.60	1.15-5.90	.022
High HB (≥ 60.7%min/h) vs high AHI (≥ 36.1 events/h)	1.72	1.04-2.85	.033

Adjusted for age, sex, BMI, type of revascularization at baseline, diabetes, former revascularization, current smoking at baseline, hypertension, history of atrial fibrillation, acute myocardial infarction at baseline, excessive daytime sleepiness, and allocation to CPAP treatment. AHI = apnea hypopnea index; CAD = coronary artery disease; HB = hypoxic burden; MACCE = major adverse cardiovascular and cerebrovascular event; NA = not applicable; RICCADSA = Randomized Intervention With CPAP in Coronary Artery Disease and Sleep Apnea.

## Discussion

In this secondary analysis of the RICCADSA cohort, high HB, but not AHI of ≥ 30 events/h, at baseline was associated with MACCEs in patients with moderate to severe OSA. Moreover, the association was strongest among participants who were untreated or nonadherent to CPAP and participants with EDS at baseline.

To the best of our knowledge, this is the first study evaluating the risk of MACCEs in patients with CAD and OSA in a revascularized CAD cohort comparing high HB with high AHI with additional subanalysis stratified by baseline sleepiness levels. Although the standard OSA metric, AHI, did not show any risk at the level of severe OSA (AHI ≥ 30 events/h) among the untreated and nonadherent patients, the high HB as a novel OSA metric clearly demonstrated a risk increase independently from age, sex, BMI, revascularization type, diabetes mellitus, current smoking, and former revascularization. Notably, compared with individuals with low AHI and low HB, only groups with high HB were at increased risk of MACCEs, whereas individuals with high AHI but low HB were not. A larger HR was seen in those with EDS than in those without EDS. No interaction was noted between high HB at baseline and EDS as well as allocation to CPAP treatment.

Because the recent randomized trials failed to show any beneficial effects of PAP treatment in intention-to-treat analyses in cohorts with cardiovascular disease and OSA, the predictive value of the AHI has been increasinly questioned.^20^ The AHI is the most frequently used standard metric to diagnose OSA (AHI ≥ 5 events/h) and quantify OSA severity.^21^ Based on this cutoff level, 84% of middle-aged men and 61% of middle-aged women (35-75 years) have OSA, which may be an overestimate.^22^ Moreover, AHI is not correlated strongly with symptoms,^23^ nocturnal oxygen desaturations, sleep fragmentation,^24^ or quality of life.^25^ For any given AHI, considerable differences exist in the degree of ventilatory deficit, HB, and arousal characteristics.^20^^,^^26^ Whether the inability to capture these interindividual OSA-related differences makes the AHI potentially a less informative metric of OSA severity has been debated.^12^ In addition to the AHI, other standard measures of OSA severity, such as the oxygen desaturation index also are used to define intermittent hypoxemia, an important consequence that seems to be responsible for most of the systemic complications of OSA.^20^

Azarbarzin et al^14^ reported first large study introducing this new novel metric that addressed the relationship of HB with cardiovascular mortality in 2019. The researchers used 2 large community-based cohort studies: the Osteoporotic Fractures in Men Study (MrOS), including 2,743 men with an average age of 76 years, and the Sleep Heart Health Study (SHHS), including 5,111 adults (53% women) with an average age of 64 years. Participants in the highest 2 HB quintiles presented a fully adjusted HR of 1.81 (95% CI, 1.25-2.62) and 2.73 (95% CI, 1.71-4.36), respectively, in the MrOS study. In the SHHS cohort, those in the highest quintile showed an HR of 1.95 (95% CI, 1.11-3.43). Moreover, in the MrOS cohort, similar results were reported regarding all-cause mortality. No association with AHI and cardiovascular mortality was reported in either cohort.^14^

Trzepizur et al^27^ recently addressed the relationship between HB and major adverse cardiovascular events and separately examined the associations between the HB and symptom subtypes in a total of 5,358 individuals with OSA (sleep clinic cohort) without previous cardiovascular events (from the Pays de la Loire Sleep Cohort).^27^ During a median follow-up of 78 months, 592 major adverse cardiovascular events were observed, and in a fully adjusted model, HB and time with < 90% oxygen saturation were the only significant predictors of major adverse cardiovascular event (HR, 1.21 [95% CI, 1.07-1.38] and 1.34 [95% CI, 1.16-1.55], respectively). Thus, these results confirmed the prognostic predictive value of HB also in individuals with OSA recruited from sleep clinics.

Blanchard et al^28^ examined the association of HB with the incidence of new cerebrovascular events using a clinical database (Pays de la Loire Sleep Cohort) linked with data from the French administrative health care database.^28^ They included 3,597 individuals followed up for almost 6 years, and a total of 83 incident cerebrovascular event were observed. The fully adjusted, log-transformed HB showed a higher prognostic value (HR, 1.28; 95% CI, 1.05-1.57) compared with other standard OSA metrics.

The data from the SHHS and MrOS studies also have been used for the association of HB and incident heart failure (HF).^29^ Azarbarzin et al^29^ included 4,881 middle-aged or older adults from the SHHS study (46% male) with an average follow-up of 10.4 years and reported 543 incident HFs. In the MrOS cohort (2,653 men with an average follow-up of 8.8 years), 145 incident HFs were observed. HB was associated strongly with incident HF in men in both the SHHS (HR, 1.18; 95% CI, 1.02-1.37) and MrOS (HR, 1.22; 95% CI, 1.02-1.45) cohorts, whereas AHI was not.

In a recent post hoc analysis of the ISAACC trial that enrolled patients with acute coronary syndrome and without excessive sleepiness, a differential pattern of response to OSA treatment was noted, with PAP depending on the baseline HB level.^16^ Pinilla et al^16^ suggested that patients with OSA with a high baseline HB not only had an increased risk of future MACCEs developing, but also exhibited a significant long-term protective effect of CPAP on cardiovascular prognosis compared with those with a low baseline HB. However, despite these promising findings, HB did not predict individuals who benefit from CPAP treatment in a post hoc analysis of the RICCADSA trial^30^; therefore, additional larger studies are needed to confirm the prognostic usefulness of HB in predicting long-term cardiovascular disease benefit of CPAP.

Our results confirm previous reports in both population and clinical cohorts supporting a prognostic value of HB regarding the adverse cardiovascular outcomes. Moreover, they suggest that this effect is strongest among the patients with CAD, OSA, and elevated ESS. A similar association between HB and MACCE risk was observed in those with and without CPAP use, although the latter estimate was nonsignificant given the smaller sample size. Notwithstanding, the fact that these results show no strong association between HB and MACCE may imply that the positive association is driven by the patients who self-selected as nonusers (and thus may be enriched by other unhealthy behaviors).

Of note, a threshold HB of > 60%min/h seems to identify patients who are at increased risk of cardiovascular morbidity and mortality.^31^^,^^32^ The median value in the current cohort shows a very similar value, and the median value also was relatively similar in ISAACC cohort (73%min/h).^16^ A plausible reason that HB is more sensitive measure of OSA-related risk is because it captures 3 dimensions of OSA metrics (ie, frequency, depth, and duration). For example, a level of 60%min/h could be reached by 20 minutes of 3% desaturation every hour or 6 minutes of 10% desaturation every hour. It is possible that treatments that improve HB without changing AHI (the frequency of events), that is, by lowering the depth and duration of desaturations, may be expected to lower the risk of MACCEs.^31^ If HB ultimately is found to be superior to AHI for cardiovascular outcomes, as several studies now suggest, then depth and duration of events will be important to track beyond frequency alone. For example, if we consider a patient with an AHI of 40 events/h and an HB of 100%min/h: if the AHI is reduced with, for example, lateral positioning to just 30 events/h (minor effect on frequency, 25% reduction), but HB is reduced to 25%min/h (major impact on depth and duration, 4-fold reduction), we may reinterpret the therapy as having potential cardiovascular risk reduction for this individual.

### Study Limitations

Our study has several limitations. First, the small sample size is a limitation especially for the analyses involving subgroups. Second, the sample included few female individuals, and we examined a sample of individuals with CAD and OSA; thus, our findings may not extend to other populations, especially female individuals. However, as noted in the Discussion, HB is demonstrated to be a significant prognostic marker for cardiovascular disease or death in both population and clinical samples. The use of a single cutoff for HB and AHI may be a limitation of this study; however, when HB was modelled as continuous variable, similar findings were observed. Nonetheless, the cutoffs for risk within each population may differ, and future studies should address the cutoffs for OSA-related hypoxemia risk of MACCEs to be used in clinical practices for prognostication. Moreover, people with an AHI of < 15 events/h on the baseline HSATs were excluded per design of the parent study. This may have put the AHI at a disadvantage when predicting future cardiovascular risk in this sample; however, our results are consistent with those of epidemiologic studies^14^^,^^29^ in which people with low AHI also were included. We also should acknowledge that there might be other potential confounders that were not included in the model. Finally, our study focused on risk stratification only, and not the effect of CPAP for secondary prevention of cardiovascular disease. Post hoc analyses of HB, akin to that in ISAACC,^16^ and prospective RCT validation are warranted to assess whether stratification of patients by HB severity can identify individuals in whom CPAP may prevent future MACCEs.

## Interpretation

High HB, but not AHI of ≥ 30 events/h, at baseline was associated with MACCEs in patients with moderate to severe OSA in this revascularized CAD cohort. Moreover, the association was strongest in the subgroup with EDS. Future RCTs are needed to assess whether targeting those with high HB improves the outcomes of PAP therapy regardless of excessive sleepiness.

## Funding/Support

This study was supported the 10.13039/501100004359Swedish Research Council [Grants 521-2011-537 and 521-2013-3439]; the 10.13039/501100003793Swedish Heart-Lung Foundation [Grants 20080592, 20090708, and 20100664]; the “Agreement concerning research and education of doctors” of Västra Götalandsregionen [Grants ALFGBG-11538 and ALFGBG-150801]; Research fund at Skaraborg Hospital [Grants VGSKAS-4731, VGSKAS-5908, VGSKAS-9134, VGSKAS-14781, VGSKAS-40271 and VGSKAS-116431]; Skaraborg Research and Development Council [Grants VGFOUSKB-46371]; the 10.13039/100002129Heart Foundation of Kärnsjukhuset; ResMed Foundation; and ResMed Ltd. ResMed Sweden provided some of the sleep recording devices and technical support. S. R. was supported by the National Institutes of Health [Grant R35 HL1358181]. A. A. is supported by the National Institutes of Health [Grants R01HL153874 and R21 HL161766] and the American Academy of Sleep Medicine [Grants 188-SR-17 and SR-2217]. A. Z. is supported by the National Heart, Lung, and Blood Institute [Grant K23HL159259]. S. S. the National Heart, Lung, and Blood Institute [Grants R01HL146697 and R01HL168067].

## Financial/Nonfinancial Disclosures

The authors have reported to *CHEST* the following: Y. P. and Y. C. report grants from ResMed Foundation. A. Z. works as a consultant for Restful Robotics, Inc., and receives grant support from ResMed Co. S. S. received grant support from Apnimed, Prosomnus, and Dynaflex; has served as a consultant for Apnimed, Nox Medical, Inspire Medical Systems, Eli Lilly, Respicardia, LinguaFlex, and Forepont; receives royalties for intellectual property pertaining to combination pharmacotherapy for sleep apnea via his institution; and is also coinventor of intellectual property pertaining to wearable sleep apnea phenotyping, also via his institution; his industry interactions are actively managed by his Institution. S. R. reports personal fees from Apnimed, Jazz Pharma, and Eli Lilly, Inc., outside the submitted work. A. A. reports grant support from Somnifix and serves as a consultant for Somnifix, Respicardia, Eli Lilly, Inspire, Cerebra, and Apnimed; Apnimed is developing pharmacological treatments for OSA. A. A.’s interests were reviewed by Brigham and Women’s Hospital and Mass General Brigham in accordance with their institutional policies.

## Supplementary Data

e-Online Data

e-Online Data

e-Online Data
