# Corrigendum to: Peker Y, Celik T, Zinchuk A, et al. Association of Hypoxic Burden With Cardiovascular Events: A Risk Stratification Analysis of the Randomized Intervention With CPAP in Coronary Artery Disease and Sleep Apnea Cohort. *CHEST*. 2025;168(6):1481-1493.

**DOI:** 10.1016/j.chest.2026.04.024

**Published:** 2026-06-09

The December 2025 article titled “Association of Hypoxic Burden With Cardiovascular Events: A Risk Stratification Analysis of the Randomized Intervention With CPAP in Coronary Artery Disease and Sleep Apnea Cohort” (*Chest*. 2025;168(6):1481-1493) contained the following errors: an incorrect affiliation was included for author Yeliz Celik, PhD.

The correct affiliation for Dr. Celik should have been listed as Koç University, Istanbul, Türkiye.

