# Supplementary material for: Association of Hypoxic Burden With Cardiovascular Events: A Risk Stratification Analysis of the Randomized Intervention With CPAP in Coronary Artery Disease and Sleep Apnea Cohort
Source: Chest. 2025 Aug 14;168(6):1481–93. doi: 10.1016/j.chest.2025.07.4081 (PMC12833478; doi:10.1016/j.chest.2025.07.4081)
Supplement: e-Online Data [file mmc1.doc]

**Late Registration Questions and Answers for the RICCADSA Trial**

1. **A detailed explanation for why the trial was registered late?**

We planned the RICCADSA trial by the end of 2004 and in early 2005, and were mainly focused on the infrastructure of the study. The date for the registration with the Swedish database was on 29 April 2005. At that time, we were unaware of the new criteria for trial registrations, and recognized these changes first when we submitted the “Rationale and Design of the RICCADSA trial” to Scandinavian Cardiovascular Journal in 2007, and completed with the registry at ClinicalTrials.gov. The rationale and design of the RICCADSA trial was published in 2009 (1).

The Swedish database, which is a registry of research grant applications, has always been accessible by public in Swedish. There was no list of the approved registries by ICMJE or WHO available during the first years, and the International Clinical Trials Registry Platform became functional first in May 2007.

1. **The reason that prompted trial registration?**

Unfortunately, as mentioned above, just unawareness of the new demands regarding an international trial registration in the early phases of the study planning.

1. **The precise date of trial registration and the date the first participant was enrolled?**

Registration with the ClinicalTrials gov was done on August 20, 2007. The first patient was enrolled in the trial on November 24, 2005.

1. **The number and percentage of patients, compared to the final sample size, enrolled prior to registration?**

In total, 167 out of 511 patients (32.7% of the final sample size) were enrolled by August 19, 2007, prior to registration with the ClinicalTrials.gov.

1. **The primary outcome/secondary outcome as stated in the trial registration?**

For the initial trial registration in ClinicalTrials gov, the outcomes were described as they were described in the manuscript in the Scandinavian Cardiovascular Journal (ref 1).

The updates were done accordingly at ClinicalTrials gov for the extension of the follow-up period and increasing the sample size after the interim-analysis in Hamburg in February 2010, which is described in the Supplemental Material of the main manuscript of the primary outcomes of the RCT-arm (ref 2).

1. **Were any data examined before registration, if so, what data?**

No data were examined before registration at the ClinicalTrials.gov on August 20, 2007. According to the initial planning, the first data for the first patient enrolled in the trial would not be collected before 3 years*, i.e.*, before November 23, 2008. The patient recruitment was ongoing, and only 1/3 of the total sample size was enrolled at the time of the trial registration at ClinicalTrials.org as mentioned above.

1. **Your assurances that no interval analysis was conducted prior to the registration of the trial?**

The first interim analysis for the main trial was done in February 2010 by Prof Karl Wegscheider and PhD Christine Eulenburg. As explained above, there was no possibility to conduct such an analysis for the primary endpoint per definition before November 23, 2008 for the first patient enrolled in the trial.

1. **Any other information you think relevant to the request?**

We think that the RICCADSA trial includes important data; we have published manuscripts including rationale/design, baseline characteristics, some cross-sectional clinical data, and the primary and secondary outcomes of the main RCT arm. These explanations have been approved by the Editors of the corresponding journals:

**Published articles from the RICCADSA Trial by May 18, 2022:**

1. Peker Y, Glantz H, Thunström E, Kallryd A, Herlitz J, Ejdebäck J. Rationale and design of the Randomized Intervention with CPAP in Coronary Artery Disease and Sleep Apnoea (RICCADSA) trial. *Scand Cardiovasc J* 2009;43:24-31.
2. Peker Y, Glantz H, Eulenburg C, Wegscheider K, Herlitz J, Thunström E. Effect of Positive Airway Pressure on Cardiovascular Outcomes in Coronary Artery Disease Patients with Non-Sleepy Obstructive Sleep Apnea: The RICCADSA Randomized Controlled Trial. *Am J Respir Crit Care Med* 2016;194:613-620.
3. Thunström E, Glantz H, Fu M, Yucel-Lindberg, Petzold M, Lindberg K, Peker Y. Increased inflammatory activity in nonobese patients with coronary artery disease and obstructive sleep apnea. *Sleep* 2015; 38:463-471.
4. Glantz H, Thunström E, Johansson MC, Wallentin Guron C, Uzel H, Ejdebäck J, Nasic S, Peker Y. Obstructive sleep apnea is independently associated with worse diastolic function in coronary artery disease. *Sleep Med* 2015; 16:160-167.
5. Glantz H, Thunstrom E, Herlitz J, Cederin B, Nasic S, Ejdeback J, Peker Y. Occurrence and predictors of obstructive sleep apnea in a revascularized coronary artery disease cohort. *Ann Am Thorac Soc* 2013; 10: 350-356.
6. Glantz H, Johansson MC, Thunstrom E, Wallentin Guron C, Uzel H, Saygin M, Herlitz J, Peker Y. Effect of CPAP on diastolic function in coronary artery disease patients with nonsleepy obstructive sleep apnea: A randomized controlled trial. *Int J Cardiol* 2017; 241:12-18.
7. Thunström E, Glantz H, Yucel-Lindberg T, Lindberg K, Saygin M, Peker Y. CPAP Does Not Reduce Inflammatory Biomarkers in Patients With Coronary Artery Disease and Nonsleepy Obstructive Sleep Apnea: A Randomized Controlled Trial. *Sleep* 2017 Nov 1;40(11).
8. Peker Y, Thunström E, Glantz H, Wegscheider K, Eulenburg C. Outcomes in coronary artery disease patients with sleepy obstructive sleep apnoea on CPAP. *Eur Resp J* 2017; Dec 7;50(6). pii: 1700749.
9. Luyster FS, Strollo P J Jr, Thunström E, Peker Y. Long-term use of continuous positive airway pressure therapy in coronary artery disease patients with nonsleepy obstructive sleep apnea. *Clin Cardiol* 2017;1–6.
10. Balcan, B., Thunström, E., Strollo Jr, P. J., & Peker, Y. Continuous positive airway pressure treatment and depression in adults with coronary artery disease and nonsleepy obstructive sleep apnea. A secondary analysis of the RICCADSA trial. *Annals of the American Thoracic Society*, 2019; 16, 62-70.
11. Baniak, L. M., Chasens, E. R., Luyster, F. S., Strollo, P. J., Thunström, E., Peker, Y. Obstructive sleep apnea and self-reported functional impairment in revascularized patients with coronary artery disease in the RICCADSA trial. *Sleep and Breathing* 2018*; 22*(4), 1169-1177.
12. Balcan B, Thunström E, Strollo PJ Jr, Peker Y. Continuous Positive Airway Pressure and Depression in Adults with Coronary Artery Disease and Nonsleepy Obstructive Sleep Apnea. A Secondary Analysis of the RICCADSA Trial. *Ann Am Thorac Soc* 2019 Jan;16(1):62-70. doi: 10.1513/AnnalsATS.201803-174OC.
13. Balcan B, Thunström E, Strollo PJ Jr, Peker Y. Determinants of depressive mood in coronary artery disease patients with obstructive sleep apnea and response to continuous positive airway pressure treatment in non-sleepy and sleepy phenotypes in the RICCADSA cohort. *J Sleep Res* 2019 Aug;28(4):e12818. doi: 10.1111/jsr.12818.
14. Balcan B, Thunström E, Yucel-Lindberg T, Lindberg K, Ay P, Peker Y. Impact of CPAP treatment on leptin and adiponectin in adults with coronary artery disease and nonsleepy obstructive sleep apnoea in the RICCADSA trial. *Sleep Med*. 2019 Nov 14;67:7-14.
15. Wallström S, Balcan B, Thunström E, Wolf A, Peker Y. CPAP and Health-Related Quality of Life in Adults with Coronary Artery Disease and Nonsleepy Obstructive Sleep Apnea in the RICCADSA Trial. *J Clin Sleep Med* 2019 Aug 12.
16. Peker Y, Thunström E, Glantz H, Eulenburg C. Effect of Obstructive Sleep Apnea and CPAP Treatment on Cardiovascular Outcomes in Acute Coronary Syndrome in the RICCADSA Trial. *J Clin Med* 2020 Dec 15;9(12):4051. doi: 10.3390/jcm9124051.
17. Celik Y, Thunström E, Strollo PJ Jr, Peker Y. Continuous positive airway pressure treatment and anxiety in adults with coronary artery disease and nonsleepy obstructive sleep apnea in the RICCADSA trial. *Sleep Med*. 2021 Jan;77:96-103. doi: 10.1016/j.sleep.2020.11.034. Epub 2020 Dec 4.
18. Zinchuk AV, Chu JH, Liang J, Celik Y, Op de Beeck S, Redeker NS, Wellman A, Yaggi HK, Peker Y, Sands SA. Physiological Traits and Adherence to Sleep Apnea Therapy in Individuals with Coronary Artery Disease. *Am J Respir Crit Care Med*. 2021 Sep 15;204(6):703-712.
19. Celik Y, Yapici-Eser H, Balcan B, Peker Y. Association of Excessive Daytime Sleepiness with the Zung Self-Rated Depression Subscales in Adults with Coronary Artery Disease and Obstructive Sleep Apnea. *Diagnostics* (Basel). 2021 Jun 28;11(7):1176. doi: 10.3390/diagnostics11071176.
20. Behboudi A, Thelander T, Yazici D, Celik Y, Yucel-Lindberg T, Thunström E, Peker Y. Association of TNF-α (-308G/A) Gene Polymorphism with Circulating TNF-α Levels and Excessive Daytime Sleepiness in Adults with Coronary Artery Disease and Concomitant Obstructive Sleep Apnea. *J Clin Med* 2021 Jul 31;10(15):3413. doi: 10.3390/jcm10153413.
21. Celik Y, Balcan B, Peker Y. CPAP Intervention as an Add-On Treatment to Lipid-Lowering Medication in Coronary Artery Disease Patients with Obstructive Sleep Apnea in the RICCADSA Trial. *J Clin Med* 2022 Jan 5;11(1):273. doi: 10.3390/jcm11010273.
22. Peker Y, Holtstrand-Hjälm H, Celik Y, Glantz H, Thunström E. Postoperative Atrial Fibrillation in Adults with Obstructive Sleep Apnea Undergoing Coronary Artery Bypass Grafting in the RICCADSA Cohort. *J Clin Med* 2022 Apr 27;11(9):2459. doi: 10.3390/jcm11092459.
23. Balcan B, Celik Y, Newitt J, Strollo PJ Jr, Peker Y. REM-Predominant Obstructive Sleep Apnea in Patients with Coronary Artery Disease. J Clin Med. 2022 Jul 28;11(15):4402. doi: 10.3390/jcm11154402.
24. Azarbarzin A, Zinchuk A, Wellman A, Labarca G, Vena D, Gell L, Messineo L, White DP, Gottlieb DJ, Redline S, Peker Y, Sands SA. Cardiovascular Benefit of CPAP in Adults with Coronary Artery Disease and OSA without Excessive Sleepiness. Am J Respir Crit Care Med. 2022 Sep 15;206(6):767-774. doi: 10.1164/rccm.202111-2608OC.
25. Özkan E, Celik Y, Yucel-Lindberg T, Peker Y. Current Smoking Determines the Levels of Circulating MPO and MMP-9 in Adults with Coronary Artery Disease and Obstructive Sleep Apnea. J Clin Med. 2023 Jun 14;12(12):4053. doi: 10.3390/jcm12124053.
26. Eulenburg C, Celik Y, Redline S, Thunström E, Glantz H, Strollo PJ Jr, Peker Y. Cardiovascular Outcomes in Adults with Coronary Artery Disease and Obstructive Sleep Apnea with vs without Excessive Daytime Sleepiness in the RICCADSA Cohort. Ann Am Thorac Soc. 2023 Jul;20(7):1048-1056. doi: 10.1513/AnnalsATS.202208-676OC.
27. Celik Y, Peker Y, Yucel-Lindberg T, Thelander T, Behboudi A. Association of TNF-α (-308G/A) Gene Polymorphism with Changes in Circulating TNF-α Levels in Response to CPAP Treatment in Adults with Coronary Artery Disease and Obstructive Sleep Apnea. J Clin Med. 2023 Aug 16;12(16):5325. doi: 10.3390/jcm12165325.
28. Peker Y, Celik Y, Behboudi A, Redline S, Lyu J, Wei Y, Gottlieb DJ, Jelic S. CPAP may promote an endothelial inflammatory milieu in sleep apnoea after coronary revascularization. EBioMedicine. 2024 Mar;101:105015. doi: 10.1016/j.ebiom.2024.105015. Epub 2024 Feb 24.

**Yüksel Peker, MD, PhD, Prof.**

**Principal Investigator**
