# Supplementary material for: Association of Hypoxic Burden With Cardiovascular Events: A Risk Stratification Analysis of the Randomized Intervention With CPAP in Coronary Artery Disease and Sleep Apnea Cohort
Source: Chest. 2025 Aug 14;168(6):1481–93. doi: 10.1016/j.chest.2025.07.4081 (PMC12833478; doi:10.1016/j.chest.2025.07.4081)
Supplement: e-Online Data [file mmc2.docx]

STROBE Statement—checklist of items that should be included in reports of observational studies

|  | Item No. | Recommendation | Page  No. |
| --- | --- | --- | --- |
| **Title and abstract** | 1 | (*a*) Indicate the study’s design with a commonly used term in the title or the abstract | Page 1 |
|  |  | (*b*) Provide in the abstract an informative and balanced summary of what was done and what was found | Page 1 |
| Introduction | | | |
| Background/rationale | 2 | Explain the scientific background and rationale for the investigation being reported | Page 4, Introduction |
| Objectives | 3 | State specific objectives, including any prespecified hypotheses | Page 5, Introduction |
| Methods | | | |
| Study design | 4 | Present key elements of study design early in the paper | Page 5, Study Design and Methods |
| Setting | 5 | Describe the setting, locations, and relevant dates, including periods of recruitment, exposure, follow-up, and data collection | Pages 4-5, Study Design and Methods |
| Participants | 6 | (*a*) *Cohort study*—Give the eligibility criteria, and the sources and methods of selection of participants. Describe methods of follow-up  *Case-control study*—Give the eligibility criteria, and the sources and methods of case ascertainment and control selection. Give the rationale for the choice of cases and controls  *Cross-sectional study*—Give the eligibility criteria, and the sources and methods of selection of participants | Pages 4-5, Study Design and Methods |
|  |  | (*b*) *Cohort study*—For matched studies, give matching criteria and number of exposed and unexposed  *Case-control study*—For matched studies, give matching criteria and the number of controls per case | N/A |
| Variables | 7 | Clearly define all outcomes, exposures, predictors, potential confounders, and effect modifiers. Give diagnostic criteria, if applicable | Pages 6-9, Study Design and Methods |
| Data sources/ measurement | 8* | For each variable of interest, give sources of data and details of methods of assessment (measurement). Describe comparability of assessment methods if there is more than one group | Pages 5-8, Study Design and Methods |
| Bias | 9 | Describe any efforts to address potential sources of bias | Page 9, Study Design and Methods – Statistical Analysis, Adjustment section |
| Study size | 10 | Explain how the study size was arrived at | Page 6, Study Design and Methods |

Continued on next page

| Quantitative variables | 11 | Explain how quantitative variables were handled in the analyses. If applicable, describe which groupings were chosen and why | Exposure, page 7, Study Design and Methods;  Outcome: page 7 in same section | |
| --- | --- | --- | --- | --- |
| Statistical methods | 12 | (*a*) Describe all statistical methods, including those used to control for confounding | Pages 8 – 9, Statistical analyses section | |
|  |  | (*b*) Describe any methods used to examine subgroups and interactions | Pages 8 – 9, Statistical analyses section | |
|  |  | (*c*) Explain how missing data were addressed | Page 9, Statistical analyses section | |
|  |  | (*d*) *Cohort study*—If applicable, explain how loss to follow-up was addressed  *Case-control study*—If applicable, explain how matching of cases and controls was addressed  *Cross-sectional study*—If applicable, describe analytical methods taking account of sampling strategy | Page 6, Study Design and Methods reference to | |
|  |  | (*e*) Describe any sensitivity analyses | N/A | |
| Results | | | |  |
| Participants | 13* | (a) Report numbers of individuals at each stage of study—eg numbers potentially eligible, examined for eligibility, confirmed eligible, included in the study, completing follow-up, and analysed | Page 10, Results; Figure 1. | |
|  |  | (b) Give reasons for non-participation at each stage | Page 10, Results; Figure 1. | |
|  |  | (c) Consider use of a flow diagram | Figure 1. | |
| Descriptive data | 14* | (a) Give characteristics of study participants (eg demographic, clinical, social) and information on exposures and potential confounders | Table 1 | |
|  |  | (b) Indicate number of participants with missing data for each variable of interest | Table 1 | |
|  |  | (c) *Cohort study*—Summarise follow-up time (eg, average and total amount) | Page 10, Results | |
| Outcome data | 15* | *Cohort study*—Report numbers of outcome events or summary measures over time | Page 11, Results | |
|  |  | *Case-control study—*Report numbers in each exposure category, or summary measures of exposure | *N/A* | |
|  |  | *Cross-sectional study—*Report numbers of outcome events or summary measures | *N/A* | |
| Main results | 16 | (*a*) Give unadjusted estimates and, if applicable, confounder-adjusted estimates and their precision (eg, 95% confidence interval). Make clear which confounders were adjusted for and why they were included | Page 11, Table 3, Results | |
|  |  | (*b*) Report category boundaries when continuous variables were categorized | Page 11, Table 3, Results | |
|  |  | (*c*) If relevant, consider translating estimates of relative risk into absolute risk for a meaningful time period | N/A | |
| Other analyses | 17 | Report other analyses done—eg analyses of subgroups and interactions, and sensitivity analyses | Pages 11-12, Results section, Figures 2 and 3. | |

Continued on next page

| Discussion | | | |  |
| --- | --- | --- | --- | --- |
| Key results | 18 | Summarise key results with reference to study objectives | Page 12, Discussion |  |
| Limitations | 19 | Discuss limitations of the study, taking into account sources of potential bias or imprecision. Discuss both direction and magnitude of any potential bias | Page 16, Discussion, Limitations section |  |
| Interpretation | 20 | Give a cautious overall interpretation of results considering objectives, limitations, multiplicity of analyses, results from similar studies, and other relevant evidence | Page 17, Discussion, Interpretation section |  |
| Generalisability | 21 | Discuss the generalisability (external validity) of the study results | Page 16, Discussion, Limitations section |  |
| Other information | |  | |  |
| Funding | 22 | Give the source of funding and the role of the funders for the present study and, if applicable, for the original study on which the present article is based | Provided in submission form and documents to CHEST |  |

*Give information separately for cases and controls in case-control studies and, if applicable, for exposed and unexposed groups in cohort and cross-sectional studies.

**Note:** An Explanation and Elaboration article discusses each checklist item and gives methodological background and published examples of transparent reporting. The STROBE checklist is best used in conjunction with this article (freely available on the Web sites of PLoS Medicine at http://www.plosmedicine.org/, Annals of Internal Medicine at http://www.annals.org/, and Epidemiology at http://www.epidem.com/). Information on the STROBE Initiative is available at www.strobe-statement.org.
