# Supplementary material for: Association of Hypoxic Burden With Cardiovascular Events: A Risk Stratification Analysis of the Randomized Intervention With CPAP in Coronary Artery Disease and Sleep Apnea Cohort
Source: Chest. 2025 Aug 14;168(6):1481–93. doi: 10.1016/j.chest.2025.07.4081 (PMC12833478; doi:10.1016/j.chest.2025.07.4081)
Supplement: e-Online Data [file mmc3.docx]

**Online Supplement**

**Hypoxic Burden but Not AHI Predicts Risk of Cardiovascular Events: A Risk Stratification Analysis of the RICCADSA Study**

**Yüksel Peker, Yeliz Celik, Andrey Zinchuk, Scott A. Sands, Susan Redline, Ali Azarbarzin**

**Methods**

**Study design and participants**

Patients were recruited from two hospitals serving a population of nearly 250,000 residing in the Skaraborg County of West Götaland, Sweden. In brief, the entire study population consisted of adult patients with angiography-verified CAD who had undergone percutaneous coronary intervention (PCI) or coronary artery by-pass grafting (CABG) within 6 months prior to study start. The CAD patients were classified as having OSA (apnea-hypopnea index [AHI] ≥ 15/h) or no-OSA (AHI < 5/h) based on a home sleep apnea test (HSAT). For the current protocol, only OSA patients with EDS (Epworth Sleepiness Scale [ESS] score ≥ 10) who were allocated to CPAP, and without EDS (ESS score < 10) who were randomized to CPAP or no-CPAP at baseline were included. The study participants were recruited between December 2005 and November 2010, and the follow-up was completed in May 2013.

**Study Oversight**

As previously described ([1](#_ENREF_1)) an Independent Clinical Event Committee (ICEC) reviewed all data obtained from patient records and death certificates by the end of May 2013, blinded to the group allocation. A Data Monitoring Board monitored a random 10% selection of the database for

clinical data and follow-ups, including CPAP usage and primary composite endpoints.

Sleep recordings

*Home sleep apnea test*

The portable, home sleep apnea testing (HSAT) was performed with the Embletta^®^ Portable Digital System (PDS) device (Embla, Broomfield, CO, USA). A nasal pressure detector was used for measurement of flow, thoraco-abdominal movement detection was conducted through two XactTrace™ inductive belts with respiratory inductance plethysmography technology, body position as well as movement detection was also provided, and a finger pulse-oximeter was used to detect heart rate and oxyhemoglobin saturation (SpO_2_). The participant's sleep time was estimated on the basis of self-reporting as well as the pattern of body movement during the HSAT recordings. Patients with an estimated sleep time of <4 hours were offered a new HSAT. Apneas were defined as an almost complete (≥90%) cessation of airflow. Hypopneas were defined as a ≥50% reduction in thoraco-abdominal movement and/or a ≥50% decrease in the nasal pressure amplitude for ≥10 seconds ([2](#_ENREF_2)). The total number of significant oxyhemoglobin desaturations (decrease of ≥4% from the immediately preceding baseline) were scored, and the oxygen desaturation index (ODI) was calculated as the number of significant desaturations per hour of estimated sleep. Events with 30-50% reduction in thoraco-abdominal movement and/or 30-50% decrease in the nasal pressure amplitude for at least 10 seconds were also scored as hypopneas, when there was an at least 4% decrease in SpO_2_ ([2](#_ENREF_2)). Patients with an apnea-hypopnea index (AHI) ≥15 per hour of estimated sleep time, independent of symptom occurrence were considered as having OSA diagnosis, and adults with an AHI 5.0-14.9 events/h were not included in the main trial in order to avoid overlapping of patients with *vs.* without OSA ([1](#_ENREF_1), [3](#_ENREF_3), [4](#_ENREF_4)). All baseline screening recordings were scored by the same observer (YP).

*Polysomnography*

All patients with CAD with a diagnosis of OSA based on the HSAT underwent unattended overnight PSG (Embla A10®, Embla, Broomfield, CO, USA) in the hospital. The PSG system included three-channel electroencephalography (C4-A1, C3-A2, CZ-A1]), two-channel electrooculography (EOG), one-channel submental electromyography (EMG), bilateral tibial EMG and two-lead electrocardiogram (ECG) in addition to the aforementioned cardiorespiratory channels for the HSAT system. PSG recordings were scored by an observer blinded to clinical data and baseline screening results from the HSATs. Obstructive events on the PSGs were scored according to the same apnea/hypopnea criteria applied for the HSAT recordings.

Epworth Sleepiness Scale

The Epworth Sleepiness Scale (ESS) questionnaire was used to evaluate subjective excessive daytime sleepiness ([5](#_ENREF_5)). The survey contains eight questions regarding the chance of dozing off under eight scenarios. Each item is scored from 0 to 3 (0 for would never doze, 1 for slight chance of dozing, 2 for moderate chance of dozing, and 3 for high chance of dozing). The ESS score ranges from 0 to 24. Excessive daytime sleepiness (EDS) was defined as an ESS score of ≥10 in the RICCADSA study.

**Group assignment, randomization, interventions, and follow-up**

Group assignment was based on the HSAT results at screening. The 1:1 random assignment of OSA patients without EDS in the RCT arm was scheduled by the sealed envelope system with a block size of 8 patients (four CPAP, four controls) stratified by gender and revascularization type (PCI/CABG). The participants were enrolled in the randomization procedure blinded to the details of the patient characteristics and comorbidity data. OSA patients with EDS were allocated to CPAP according to the clinical routines. All patients assigned to CPAP treatment were informed about the technical procedure and provided with an auto-titrating CPAP device (S8^®^, or S9^®^; ResMed, Sydney, Australia) and a nasal or full-face mask and humidifier by trained staff at the study center. All participants assigned to CPAP were instructed to use the device at home every night for at least 4 hours every night, contacted by telephone after one week and given a check-up in the clinic after 1 month, 3 months, 6 months, 1 year, and then yearly to end of the main study, according to the study protocol. All patients received similar support by the research and clinical teams over the period of follow-up. A new HSAT was conducted in patients at 3 and 12 months, and annually thereafter (with CPAP for the treatment groups).

**Adherence to CPAP**

OSA patients who were allocated to CPAP, brought their devices to the clinic at each scheduled follow-up visit. Monitoring settings, hours of CPAP use per night as well as CPAP days under certain periods were obtained from the machines’ internal clocks. In addition, pressure level, mask leak and residual AHI measures were noted. All necessary adjustments of the CPAP device and mask fittings were done according to clinical routines by the sleep medicine unit staff.

**Cardiovascular endpoint criteria**

As previously described ([1](#_ENREF_1), [4](#_ENREF_4)), an Independent Clinical Event Committee (ICEC) reviewed all data obtained from the medical records and death certificates by the end of May 2013, blinded to group allocation. In summary, overall mortality was based on the death certificates. Cardiovascular mortality was defined as death from any of the following: myocardial infarction, stroke (cerebral haemorrhage or cerebral infarction), ruptured aortic aneurysm (thoracic or abdominal), heart failure (as determined by the treating physician), sudden death with no cause other than presumed cardiac (malignant arrhythmias), death during or within 28 days of CABG or PCI, and pulmonary embolism. A more detailed description of the comorbidities have been published previously ([1](#_ENREF_1), [4](#_ENREF_4)).

**Data collection and analysis**

The primary outcome variables were documented prospectively and were not subject to observer bias. Baseline comorbidity data, results of sleep recordings, and CPAP compliance data were prospectively recorded in separate files at a specific server of the study hospital by clinical and research nurses blinded to study group allocation (with or without EDS).

**Results**

**Categorization of deceased cases within 12 months**

During the follow-up period, 6 patients died within 1 year (3 with EDS, 2 without EDS randomized to CPAP, 1 without EDS randomized to no-CPAP). The three patients with EDS were non-adherent at earlier time points and counted as non-compliant at 1 year. The patient without EDS who was randomized to no-CPAP was included in the no-CPAP/non-adherent group. From the two without EDS who were randomized to CPAP, one was not compliant earlier and counted as non-adherent at year 1, the other one was adherent at earlier visits and counted as adherent at the 12-month follow-up.

**REFERENCES**

1. Peker Y, Glantz H, Eulenburg C, Wegscheider K, Herlitz J, Thunstrom E. Effect of Positive Airway Pressure on Cardiovascular Outcomes in Coronary Artery Disease Patients with Nonsleepy Obstructive Sleep Apnea. The RICCADSA Randomized Controlled Trial. *Am J Respir Crit Care Med* 2016; 194: 613-620.

2. Sleep-related breathing disorders in adults: recommendations for syndrome definition and measurement techniques in clinical research. The Report of an American Academy of Sleep Medicine Task Force. *Sleep* 1999; 22: 667-689.

3. Peker Y, Glantz H, Thunstrom E, Kallryd A, Herlitz J, Ejdeback J. Rationale and design of the Randomized Intervention with CPAP in Coronary Artery Disease and Sleep Apnoea--RICCADSA trial. *Scand Cardiovasc J* 2009; 43: 24-31.

4. Peker Y, Thunstrom E, Glantz H, Wegscheider K, Eulenburg C. Outcomes in coronary artery disease patients with sleepy obstructive sleep apnoea on CPAP. *Eur Respir J* 2017; 50.

5. Johns MW. A new method for measuring daytime sleepiness: the Epworth sleepiness scale. *Sleep* 1991; 14: 540-545.
